# Mitochondrial Oxygen Monitoring During Surgical Repair of Congenital Diaphragmatic Hernia or Esophageal Atresia: A Feasibility Study

**DOI:** 10.3389/fped.2020.00532

**Published:** 2020-09-02

**Authors:** Sophie A. Costerus, Mark Wefers Bettink, Dick Tibboel, Jurgen C. de Graaff, Egbert G. Mik

**Affiliations:** ^1^Department of Pediatric Surgery, Erasmus University Medical Center-Sophia Children's Hospital, Rotterdam, Netherlands; ^2^Department of Anesthesiology, Erasmus University Medical Center, Rotterdam, Netherlands

**Keywords:** mitochondria, oxygen, neonate, surgery, monitoring

## Abstract

Current monitoring techniques in neonates lack sensitivity for hypoxia at cellular level. The recent introduction of the non-invasive Cellular Oxygen METabolism (COMET) monitor enables measuring *in vivo* mitochondrial oxygen tension (mitoPO_2_), based on oxygen-dependent quenching of delayed fluorescence of 5-aminolevulinic acid (ALA)-enhanced protoporphyrin IX. The aim is to determine the feasibility and safety of non-invasive mitoPO_2_ monitoring in surgical newborns. MitoPO_2_ measurements were conducted in a tertiary pediatric center during surgical repair of congenital diaphragmatic hernia or esophageal atresia. Intraoperative mitoPO_2_ monitoring was performed with a COMET monitor in 11 congenital diaphragmatic hernia and four esophageal atresia neonates with the median age at surgery being 2 days (IQR 1.25–5.75). Measurements were done at the skin and oxygen-dependent delayed fluorescence was measurable after at least 4 h application of an ALA plaster. Pathophysiological disturbances led to perturbations in mitoPO_2_ and were not observed with standard monitoring modalities. The technique did not cause damage to the skin, and seemed safe in this respect in all patients, and in 12 cases intraoperative monitoring was successfully completed. Some external and potentially preventable factors—the measurement site being exposed to the disinfectant chlorohexidine, purple skin marker, or infrared light—seemed responsible for the inability to detect an adequate delayed fluorescence signal. In conclusion, this is the first study showing it is possible to measure mitoPO_2_ in neonates and that the cutaneous administration of ALA to neonates in the described situation can be safely applied. Preliminary data suggests that mitoPO_2_ in neonates responds to perturbations in physiological status.

## Introduction

Major (non-cardiac) neonatal surgery is challenging for clinicians. The neonatal homeostasis is a frail equilibrium and is highly affected by general anesthesia and surgical manipulation (1, 2). The anesthesiologist aims to monitor the physiology with the help of the heart rate, invasive blood pressure, saturation, end-tidal carbon dioxide, skin perfusion, urine output, and serum lactate. These broad range of monitoring modalities are used as surrogate of end-organ perfusion with adequate oxygen transport as a prime goal. To date, the optimal blood pressure in neonates for adequate perfusion of peripheral and cerebral tissue is unknown. Invasive techniques available for effective monitoring of the circulation/cardiovascular system are seldom used due to technical restraints in neonates or are simply not feasible during neonatal surgery (3). Yet, the incidence of brain injury after (non-cardiac) neonatal surgery is increasingly reported (4, 5) as well as altered long–term neurodevelopmental outcomes (6–9). Several factors are thought to contribute to the postoperative brain injury, including alterations in the perioperative neonatal hemodynamics.

Adequate oxygen supply to tissues is of pivotal importance. A non-invasive, bedside monitoring modality for cellular oxygenation could provide direct information about oxygen transport. This allows clinician to adjust their management on actual measurements of tissue perfusion and oxygenation instead of systemic circulatory measures. In this light, monitoring of cellular oxygenation has been suggested to be beneficial during neonatal-cardiac surgery due to the highly affected hemodynamics (10). Yet, major non-cardiac congenital anomalies which requires surgery within the 1st days causes alterations in the neonatal physiology as well (4, 7). The recent introduction of the non-invasive Cellular Oxygen METabolism (COMET) monitor (Photonics Healthcare B.V., Utrecht, The Netherlands) makes it possible to measure *in vivo* mitochondrial oxygen tension (mitoPO_2_). Although mitochondrial oxygen sensing has been recognized as a promising technique for pediatric ICU and anesthesia (11, 12), until now reported use has been limited to adults (13–16). The present study tests feasibility and safety of intraoperative use of COMET monitoring in infants for the first time.

The COMET monitor measures mitoPO_2_ by means of oxygen-dependent quenching of delayed fluorescence (17). Green pulsed laser excitation of protoporphyrin IX (PpIX) leads to a relatively long-lived red-light emission, called “delayed fluorescence.” The intensity of the delayed fluorescence decays with an oxygen-dependent lifetime, meaning more oxygen results in a shorter lifetime and *vice versa*. PpIX is the final precursor of heme in the heme-biosynthetic pathway, synthesized inside the mitochondria. Under normal (non-sensitized) conditions PpIX concentrations in human skin are very low and non-detectable with COMET. Administration of 5-aminolevulinic acid (ALA) increases mitochondrial PpIX concentrations and ensures the mitochondrial origin of the delayed fluorescence signal (15). Therefore, to enable measurements with the COMET monitor, ALA needs to be applied on skin to induce PpIX, the latter acting as mitochondrially located oxygen-sensitive dye (17, 18).

ALA is registered for use in adults, for example for photodynamic therapy in dermatologic pathology (19, 20) and to visualize brain tumors during fluorescence-guided surgery (21, 22) and was not used in pediatric patients until recently. Research with cutaneous ALA administration up to 354 mg in infants of 5 years and older reported no side effects (23). Oral administration of 20 mg/kg ALA in infants of 1 year and older showed a transient increase of alanine aminotransferase (24–26). Rarely, the administration of 5-aminolevulinic acid led to an allergic reaction, in here contact dermatitis are the only reported allergies (27). Therefore, we assumed the safety on a systemic level of a very low dosage of ALA—8 mg—on the skin of neonates, providing an opportunity to use COMET monitoring in neonates for the first time. Primary outcomes of this study were feasibility and safety, especially local (photo)toxicity, of cutaneous ALA administration in combination with using the COMET monitor in neonates perioperatively. A secondary outcome was preliminary evaluation of anesthesiologic and surgical procedures influencing mitoPO_2_.

## Materials and Methods

The institutional research board approved a feasibility study of 15 neonates (MEC 2017-145).

After obtained informed consent from both parents, measurements were performed during surgical treatment of neonates with congenital diaphragmatic hernia (CDH) or esophageal atresia (EA). Surgery took place in the operating theater, unless the neonate was on extracorporeal membrane oxygenation (ECMO), in which case the surgery was performed in the pediatric intensive care unit due to logistics.

In this study the feasibility was defined as the possibility of priming the skin with ALA and to measure mitoPO_2_ in neonates. The safety was defined as (the lack of) any adverse event of the skin after cutaneous administration of ALA and measurement with COMET until 48 h after the COMET-skin sensor was removed.

**Figure 1:**
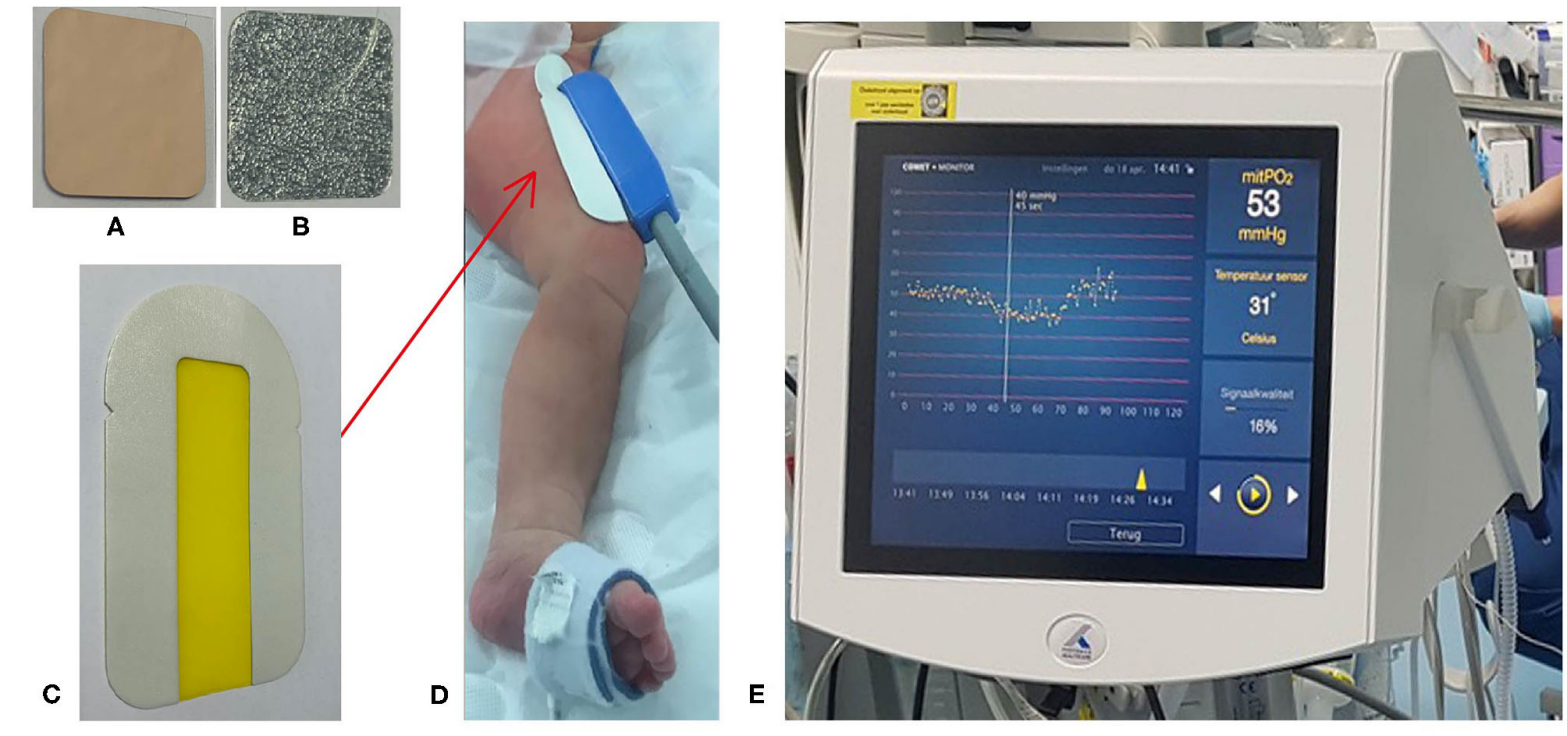
The ALA plaster with the aluminum cover **(A)** and the ALA side **(B)**, double-sided tape **(C)** which is used for the application of the COMET-skin sensor (red arrow) on the frontal side of the upper leg **(D)** and the Cellular Oxygen METabolism (COMET) monitor **(E)**.

An Alacare^®^ plaster has a square format of 2 by 2 cm and contains 2 mg per cm^2^ ALA (Alacare, photonamic, Pinneberg, Germany). The plaster is covered by an aluminum layer to protect the primed skin to light exposure (Figure 1) (28). The plaster was applied in the pediatric intensive care unit (ambient temperature of ~22°C) on the skin on the frontal side of the upper leg for at least 4 h before starting the measurement. Research in adults showed that a priming time of 4 h or more was needed to synthesize the suitable concentration of PpIX to enables measurements of mitoPO2 in the skin (15). The same minimal priming time was maintained in this study.

The COMET-skin sensor has a biocompatible housing of 7 × 2 × 2 cm. The skin sensor was placed on the primed skin and was attached to the skin by a double-sided plaster provided by the COMET manufacturer (Figure 1). The influence of light on the primed skin during the application of the COMET-skin sensor was minimized by turning off the surgical luminaires/lamps. After the application of the skin sensor, the biocompatible housing was covered with aluminum foil.

Continuous registration of routine vital parameters, regional cerebral oxygenation (rSO_2_) (INVOS^TM^ 5100C) and mitochondrial saturation (COMET) were obtained and stored for off-line analyses. Sampling rate of the vital parameters was every second, rSO_2_ every 6 s and mitochondrial oxygen tension (mitoPO_2_) every 60 s. Intraoperative management was registered in our Patient Data Management System. Patients received general anesthesia with sevoflurane/midazolam, rocuronium and fentanyl. MitoPO_2_ measurements started before surgery and continued until after surgery. After completion of the measurement the primed skin was shielded against light with an aluminum plaster for 48 h. This is based on the pharmacological characteristics of ALA. The mean half-life fluorescence clearance of PpIX is 30 ± 10 h.

## Results

Informed consent was obtained in 11 CDH and 4 EA patients. Intraoperative measurements were performed in all 15 included neonates. Neonates had a median gestational age of 38 weeks (IQR 37.7–40.2), a median birth weight of 3,000 grams (IQR 2,400–3,340) and a median age at surgery of 2 days (IQR 2–5.5). Median duration of the surgical procedure was 106 min (IQR 95–116) and two patients received surgical repair of CDH on ECMO in the pediatric intensive care unit (Table 1). Median skin priming time with ALA was 7 h 45 m (IQR 6 h 50 m−12 h 0 m). Twelve out of 15 measurements were successful with a median duration of the MitoPO_2_ measurement of 116 min (IQR 98–133) (Table 1). The first measurement failed due to the radiant warmer (infra-red light), the second due to pink chlorohexidine-alcohol disinfectants and the third due to purple skin marker on the primed skin.

**Table 1:** Patient demographics.

***n* = 15**	**Median (IQR)**
Male gender, *n* (%)	8 (53%)
Gestational age, wk	38.1 (37.7–40.2)
Birth weight, grams	3,000 (2,400–3,340)
Age at surgery, days	2 (2–5.5)
Duration of surgery, min	106 (95–116)
Priming time skin, min	465 (413–720)
Duration MitoPO_2_ measurement	116 (98–133)
**Surgical approach**	
Thoracoscopy, *n* (%)	5 (33%)
Thoracotomy, *n* (%)	2 (13%)
Laparotomy, *n* (%)	8 (53%)
Surgery during ECMO, *n* (%)	2 (13%)

In the 12 successful measurements (Table 2) the mitoPO_2_ interquartile range at start of the measurement was 51–60 mmHg. In all neonates the skin was examined on regular timepoints; after removing the ALA plaster after priming of the skin, directly after removing the COMET-sensor, at 24 and 48 h after removing the COMET-sensor. No adverse events such as erythema or other signs of an irritated skin were observed.

**Table 2:** Median and IQR values of the 12 successfully obtained measurements.

	**HR**	**MABP**	**Saturation**	**rSO_**2**_**	**MitoPO_**2**_**
Start	133 (113–142)	41 (37–44)	96 (94–97)	87 (66–93)	58 (51–60)
measurement					
+10 min	130 (112–146)	48 (40–53)	94 (91–97)	83 (69–92)	57 (55–64)
+20 min	133 (118–140)	49 (40–62)	96 (93–97)	88 (69–93)	54 (53–63)
+30 min	133 (122–151)	47 (44–49)	95 (94–97)	81 (74–93)	53 (49–60)
+40 min	146 (135–160)	42 (35–46)	92 (90–97)	79 (70–88)	53 (52–56)
+50 min	144 (137–156)	41 (35–48)	95 (91–99)	82 (72–89)	50 (48–54)
+60 min	149 (137–164)	43 (39–45)	97 (91–99)	88 (77–95)	51 (49–54)
+70 min	154 (136–166)	45 (40–48)	96 (92–97)	87 (65–94)	52 (49–58)
+80 min	150 (137–168)	45 (35–46)	96 (95–99)	86 (71–95)	52 (47–59)
+90 min	151 (133–168)	42 (37–48)	97 (91–99)	83 (67–94)	53 (52–59)
+100 min	157 (124–163)	42 (39–45)	96 (92–99)	78 (65–91)	51 (50–63)
+110 min	133 (121–168)	46 (42–52)	97 (93–99)	84 (68–91)	53 (50–64)
+120 min	137 (127–171)	42 (37–52)	96 (92–99)	77 (74–91)	48 (45–53)

**Figure 2:**
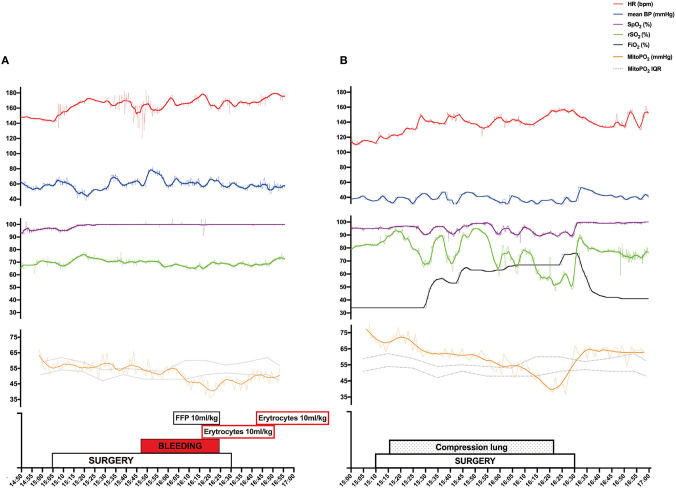
Surgical repair of congenital diaphragmatic hernia during EMCO **(A)** Surgical repair of esophageal atresia **(B)**.

Two cases illustrate fluctuations in mitoPO_2_ in relation to surgical and anesthetic actions. Case 1 (Figure 2A) is a female neonate, gestational age 37 weeks, birth weight 2,500 grams, with CDH requiring veno-arterial ECMO treatment due to therapy-resistant pulmonary hypertension. Surgical treatment was on day 8 of life, during ECMO. Priming of the skin with ALA was 6 h. During surgery bleeding intercostal arteries caused significant blood loss. Vital parameters and rSO_2_ remained unchanged, but mitoPO_2_ decreased from 62 mmHg at start surgery to 36 mmHg (a reduction of 42%) during blood loss and partially recovered after supplementation with erythrocyte transfusion with a mitoPO_2_ up to 53 mmHg at the end of the surgery.

Case 2 (Figure 2B) is a male neonate, gestational age 34 weeks, birth weight 1,950 grams, with EA type C with a trachea-esophageal fistula. Surgical repair took place on day 1 of life. Skin priming time with ALA was 8 h. The patient was positioned on the left side during surgery. Surgical compression of the lung caused hypoxia which required increasing FiO_2_ from 35 to 75% to maintain peripheral saturation between 90 and 95%. Blood pressure and heart rate remained stable, rSO_2_ responded on the increased FiO_2_ firstly, but mitoPO_2_ decreased soon after the compression started and continues to decrease from 69 mmHg at start surgery to 37 mmHg (a reduction of 47%) and restored within minutes after manipulation of the lung was finished with a mitoPO_2_ up to 62 mmHg at the end of the surgery.

## Discussion

This is the first study showing feasibility of mitoPO_2_ measurements in neonates, and importantly, in a clinically relevant high-risk perioperative setting. Measurements with the COMET monitor proved feasible and safe in terms of local damage to the skin. Furthermore, pathophysiological disturbances led to perturbations in mitoPO_2_. In 12 out of 15 patients mitoPO_2_ measurements were successful. Failures were caused by external and potentially preventable factors, disabling detection of an adequate delayed fluorescence signal. In one case infrared warming lamp heat or radiation interfered with the priming of the skin with ALA. Aluminum foil is a strong infrared reflector and was successfully used to shield the ALA plaster against infrared radiation during priming of the skin in the following cases. In the two other failed cases colored substances on the skin interfered with measurements, chlorohexidine with pink pigment and skin marker are both significant sources of delayed fluorescence and thereby potent disturbers of the mitochondrial PpIX light emission.

Safety of ALA administration with Alacare plasters was a major concern for the ethics committee due to the off-label use of ALA for measuring mitoPO_2_ with the COMET. The reaction of the neonatal skin on ALA administration was unknown and consequently we only obtained approval to perform this feasibility and safety study. ALA makes the skin sensitive for light, consequently it is frequently used for photodynamic therapy in different sorts of dermatologic pathology. In children of 5 years and older, the administration of ALA up to 354 mg, which is over 40 times higher than de 8 mg ALA that was applied on the skin in this study, did not have any side effects (23). Oral administration of 20 mg/kg ALA in infants of 1 year and older showed a transient increase of alanine aminotransferase (24–26). Systemic effects of topical/local administration of ALA on the skin have not been reported and in this study, we focused on potential local side effects in neonatal skin.

There is a risk for erythema and burns when the skin is exposed to (day)light after the administration of ALA. Therefore, precautionary measures were taken to shield the skin for light for 48 h after the measurement with the COMET was ended and the skin sensor was removed. In none of the cases local damage or irritation of the skin was observed, so the combination of ALA-plaster and COMET measurements seems safe.

The pharmacokinetic properties of topical ALA administration with Alacare in neonates are unknown, but in adults the reported skin priming time with ALA takes 4 till 8 h (13). In this study, the same priming times were maintained for neonates. In a following efficacy study, the power calculation/sample size will be focused on validating mitoPO_2_ measurements in neonates and analyzing the ideal priming time of the neonatal skin. This will create insight in the reaction of the skin to the application of ALA in term and preterm neonates.

For this study two major non-cardiac congenital anomalies were included: congenital diaphragmatic hernia (CDH) and esophageal atresia (EA). These congenital anomalies were chosen to be eligible because major surgery is required within the 1st days of life and postoperative brain injury are reported in children with these congenital anomaly (4, 7). CDH neonates suffer from lung hypoplasia and abnormal morphology of the pulmonary vasculature which results in respiratory insufficiency and severe (therapy-resistant) pulmonary hypertension (29, 30). CDH neonates are a challenge for clinicians to manage due this altered physiology. In EA neonates, the physiology is less affected by the congenital anomaly itself, but requires complex surgery with major intrathoracic manipulation which highly affects the neonatal physiology (31). In these children, our preliminary results suggest that monitoring mitochondrial oxygenation might register changes in neonatal physiology which could not have been observed using standard monitoring devices. Clearly, further research into the clinical usability of COMET is warranted but seems justified based on this pilot. Although this was only a feasibility and safety study, these results confirm that mitochondrial hypoxia may occur without clear signs of central hypoxia and are in line with previous research in animals and humans (32–35). A piglets study demonstrated cutaneous mitoPO_2_ changed earlier than MABP and lactate during ongoing hemodilution (32). In a sepsis rat model as well as in rats with induced right ventricular failure due to pulmonary arterial hypertension, mitoPO_2_ proved an additional parameter monitoring physiological changes (33, 34). The clinical prototype of the COMET was tested in healthy volunteers and showed measuring mitochondrial oxygenation and oxygen consumption in humans (13). Previous reports demonstrated the intraoperative use of COMET in adults (15) and also demonstrated that mitoPO_2_ measurements are not limited to the skin (35). The first study using COMET during upper gastro-intestinal endoscopy showed it is technically feasible and safe (35).

Adequate oxygen supply to tissues is of pivotal importance to sustain mammalian life. Aerobic metabolism is maintained through inhalation of air in the lungs and subsequent transport of the absorbed oxygen to tissues *via* the circulation. The flow of hemoglobin-bound oxygen through the macro- and microcirculation and diffusion of molecular oxygen into the tissue cells brings oxygen to the mitochondria. In the mitochondria, oxygen is used in oxidative phosphorylation in order to efficiently produce adenosine triphosphate (ATP) that acts as the energy source for many cellular processes. Furthermore, mitochondria are essential for homeostasis of the cell, they play a major role in (programmed) cell death (apoptosis). Opening of the mitochondrial permeability transition pore, as a result of a stressful stimulus such as calcium or reactive oxygen species overload, leads to loss of the mitochondrial membrane potential (36). The collapse of the membrane potential results in ATP depletion and necrosis (37), and the release of mitochondrial content such as cytochrome c leads to apoptosis (38). A correlation to outcome after perturbations in cellular oxygenation have not yet been shown, but it could be used as an early warning sign. Importantly, in both a preclinical (32) and clinical setting (15) mitoPO_2_ provided different information than hemoglobin saturation-based techniques like near- infrared spectroscopy (NIRS). Although visible light spectroscopy and near-infrared spectroscopy failed to show any response on a perturbation, mitoPO_2_ clearly dropped. This was observed during hemodilution in piglets, where mitoPO_2_ was measured simultaneously with tissue oxygen saturation on the thoracic wall. The mitoPO_2_ decreased after the hemoglobin dropped below a threshold, but tissue oxygen saturation, which was measured with NIRS, did not (32).

We previously published a clinical example in which mitoPO_2_ showed a different response than microvascular hemoglobin-saturation. During peripheral vasoconstriction, which was induced by the administration of clonidine, microvascular flow, and velocity parameters measured with laser-doppler decreased both. The venous-capillary oxygen saturation did not decrease, however, mitoPO_2_ in the skin measured by COMET decreased along with the decrease in flow and velocity (15). While mitoPO_2_ and microvascular flow provided similar information here, we expect additional value of mitoPO_2_ measurements in clinical situations in which microvascular shunting (39) and loss of hemodynamic coherence occur (40), for example in sepsis and hemodilution. During sepsis microcirculatory dysfunction occurs which causes shunting and loss of the coherence between blood flow and tissue oxygenation. Here microvascular, and ultimately mitochondrial, oxygen measurements can be of additional value (39). The same holds true during a hyperdynamic circulation due to hemodilution, causing erythrocytes to pass too quickly through the microcirculation. This phenomenon is referred to as functional shunting and involves the inability of hemoglobin to off-load oxygen fast enough to the tissues as it passes through the microcirculation, causing cellular hypoxia while hemoglobin saturation is normal or increased (40, 41).

In this study we found baseline mitoPO_2_ values in the range of 51–60 mmHg. In a previous study in healthy volunteers we reported mean mitoPO_2_ to be 44 mmHg, and in a very recently published study in critical care patients mean mitoPO_2_ was reported to be around 60 mmHg (42). Such relatively high values match well with other oxygen measurements in skin (43). The differences between the studies could well be attributed to factors like skin temperature, filling status of the patient, and use of sedation/anesthesia, since such factors are known to influence skin perfusion. Clinical data until now are scarce and normal values for mitoPO_2_ remain to be determined, as well as the influence of patient factors (such as age) and clinical circumstances. Although we do think mitochondrial oxygen tension is in general higher than anticipated (12), the reader should realize that mitoPO_2_ in other organs and tissues is likely to differ. Differences in tissue oxygen levels exist between organs, tissues, and tissue compartments (43) and metabolic activity (for example muscle contraction) is also of influence.

To date, clinicians are in the dark about the effect of the altered neonatal (patho)physiology during major high-risk surgery on cellular oxygenation. In the past the focus was to optimize macrohemodynamics although the microcirculation has been increasingly recognized as an import variable in the critically ill neonate (44). To measure tissue oxygenation, a modality based on the principle of near infrared spectroscopy (NIRS) became popular. The optode of the NIRS emits near-infrared light, which easily penetrates biological tissue at a depth of ~2–3 cm (45, 46). It measures the oxygenation of a combination of 75% venous, 20% arterial, and 5% capillary blood, but does not provide information about the oxygen concentration at cellular level. Unfortunately, the clinical use of additional monitoring with NIRS have not been established yet (47). The COMET allows us to look at oxygen availability at a cellular level. The neonatal skin is an ideal target organ for COMET measurements. It is the biggest organ in neonates and has a relative bigger surface and is more vascularized compared to adults. Skin blood circulation is very sensitive to changes in vascular resistance and blood pressure (48), potentially making the skin a good indicator for the (general) cardiopulmonary status of the neonate.

Compared to interstitial measurements with for example oxygen electrodes COMET has some distinct advantages, such as no need for calibration, non-destructiveness (no need for needle placement), well-defined measurement compartment and very fast response time (no need for signal integration over longer periods of time). A disadvantage of the COMET technique is the necessary priming with ALA. Although previous studies in adults and this study in neonates, show that with some precaution's application of ALA to the skin can be done without harm, it requires planning and currently prevents its use in emergency situations. In elective situations in the operating room and for use in the intensive care this proved not a major issue.

In conclusion, this is the first study showing it is possible to measure mitoPO_2_ in neonates and that the cutaneous administration of ALA to neonates in the described situation can be safely applied. Preliminary data suggests that mitoPO_2_ in neonates responds to perturbations in physiological status. The added value of mitochondrial measurements for clinical decision making remains to be determined in future studies.

## Data Availability Statement

All datasets presented in this study are included in the article/supplementary material.

## Ethics Statement

The studies involving human participants were reviewed and approved by Erasmus Medical Center. Written informed consent to participate in this study was provided by the participants' legal guardian/next of kin.

## Author Contributions

All authors had a substantial contribution to conception and design, acquisition of data, analysis, interpretation of data, participated in drafting the article or revising it critically for important intellectual content, and gave final approval of the version to be submitted.

## Conflict of Interest

EM is founder and shareholder of Photonics Healthcare B.V., the company that developed and commercializes the COMET monitor. Photonics Healthcare B.V. holds the exclusive licenses to several patents regarding this technology, filed and owned by the Academic Medical Center in Amsterdam and the Erasmus University Medical Center Rotterdam, the Netherlands. The remaining authors declare that the research was conducted in the absence of any commercial or financial relationships that could be construed as a potential conflict of interest.
